# Supplementary material for: Nutrition Therapy in Critically Ill Patients with Obesity: An Observational Study
Source: Nutrients. 2025 Feb 19;17(4):732. doi: 10.3390/nu17040732 (PMC11858426; doi:10.3390/nu17040732)
Supplement: Supplementary file 1 [file nutrients-17-00732-s001.zip › nutrients-3422449-supplementary.pdf]

Table S1. STROBE (Strengthening the Reporting of Observational Studies in Epidemiology Statement) checklist of items that have been included and reported along the manuscript.

Item	How STROBE the recommendation has been fulfilled
1. Title & abstract	The observational nature of the research is reported in the title and abstract.
	An informative and balanced summary of what was done and what was found during the research process is reported in the abstract briefly.
2. Introduction: rationale	The scientific background and rationale for the investigation being reported has been developed based on epidemiological and clinical importance, as well as the controversy that represent the recommendations about nutrition therapy in critically ill patients with obesity.
3. Introduction: objectives	Specific objectives (main and secondary) have been stated at the end of the introduction section.
4. Methods: study design	key elements of study design, such as the observational and the multivariate nature of the study, development and organizational issues are described within the text.
5. Methods: setting	The setting, locations, participants, and periods of recruitment and data collection are fully reported.
6. Methods: participants	Inclusion and exclusion criteria to show specifically the sources and methods of selection of patients included in the database are included.
7. Methods: Variables, data sources, measurement	We have clearly defined variables included in the database and outcomes. Definitions of those specific recorded variables or references related with those variables have been provided when needed (e.g., high gastric residual volume). We have also described sources of data.
8. Methods: Bias & study size	We have briefly described how study sites have been selected and further expand how selection criteria were performed to address potential sources of bias and achieve the objectives of the study. How the study size was arrived at the number of patients recruited is described in a flow chart (Figure 1).
9. Methods: quantitative variables	The explanation of how quantitative variables were chosen, which subgroups were selected, and how and why were handled for analyses are fully described.
10. Statistical methods	All statistical methods, including those used to control for confounding, methods used to examine subgroups and interactions, how missing data were handled during analysis and if any data transformations were applied (when assumptions were violated), are described in order to add more transparency the whole research process.
11. Results: Participants	Numbers of patients included at each stage of study, together with reasons for non-participation at each stage, are provided throughout a flow diagram.
12. Results: Descriptive & outcome data	Characteristics of study participants (e.g.; clinical comorbidities on ICU admission), as well as numbers of outcome events are provided.
13. Main Results	Main observed results were provided in tables and along the text with univariate analyses and multivariate analyses, together with their confounder-adjusted estimates and their precision (i.e., 95% confidence interval), with a clear explanation of which confounders were adjusted for and why they were included.
14. Discussion: Key results, interpretation & generalisability	The key results are summarized and described with reference to study aims and objectives. A cautious overall interpretation of results considering the whole scenario and nature of the present research (i.e., objectives, limitations, statistical analyses, results from similar studies and other relevant evidence) has been given within the Results section. The external validity of the present

	research is also addressed.
15. Discussion: Limitations	Limitations of the study, taking into account sources of potential bias, are widely discussed for an appropriate interpretation of the results of the research.
16. Funding	The source of funding and their role have been given for the present study.

Table S2. General characteristics, nutritional therapy, and outcomes of patients admitted to the ICU based on body mass index subgroup.

		All patients n = 525	Normal n = 165	Overweight n = 210	Obese n = 150	p-Value
**Baseline characteristics & comorbidities**						
Age, years, mean ± SD		61.5 ± 15	58.8 ± 16.5	62.8 ± 14.7	62.7 ± 13.5	0.05
Gender, male patients, n (%)		67.2% (353)	64.8% (107)	74.8% (157)	59.3% (89)	**0.003[B]**
Hypertension, n (%)		43.6% (229)	33.9% (56)	41.9% (88)	56.7% (85)	**0.01[A, B]**
Diabetes mellitus, n (%)		25% (131)	21.2% (35)	20% (42)	36% (54)	**0.001[A, B]**
COPD, n (%)		16.6% (87)	13.9% (23)	18.1% (38)	17.3% (26)	0.53
AMI, n (%)		14.1% (74)	8.5% (14)	16.7% (35)	16.7% (25)	**0.04 [B]**
Chronic liver disease, n (%)		4.8% (25)	3.6% (6)	4.3% (9)	6.7% (10)	0.41
Chronic renal failure, n (%)		9.9% (52)	7.3% (12)	10.5% (22)	12% (18)	0.35
Immunosuppression, n (%)		11.6% (61)	15.2% (25)	11.9% (25)	7.3% (11)	0.09
Neoplasia, n (%)		20.6% (108)	24.2% (40)	19.5% (41)	18% (27)	0.11
Type of patient	Medical, n (%)	63.8% (335)	65.5% (108)	62.9% (132)	63.3% (95)	0.81
	Trauma, n (%)	12.6% (66)	10.9% (18)	15.2% (32)	10.7% (16)	0.75
	Surgery, n (%)	23.6% (124)	23.6% (39)	21.9% (46)	26% (39)	0.67
**Prognosis ICU scores & nutrition status on ICU admission**						
APACHE II, mean ± SD		20.3 ± 7.9	19.7 ± 7.6	20.1 ± 7.5	21.2 ± 8.5	0.18
SAPS II, mean ± SD		49.1 ± 17.7	47.2 ± 17.1	48.6 ± 17.2	52 ± 18.7	0.12
SOFA, mean ± SD		7.2 ± 3.4	6.8 ± 3.5	7.2 ± 3.3	7.6 ± 3.5	0.54
Malnutrition (based on SGA), n (%)		41% (215)	52.7% (87)	37.1% (78)	33.3% (50)	**0.01 [B]**
mNUTRIC score, mean ± SD		4.2 ± 2.1	4.1 ± 2.1	4.2 ± 2.2	4.4 ± 2	0.28
Patient at risk (based on mNUTRIC), n (%)		44.4% (233)	39.4% (65)	45.2% (95)	48.7% (73)	0.22
**Characteristics of Medical Nutrition Therapy**						
Time of MNT initiation, h, mean ± SD		37.7 ± 33.3	35.1 ± 31.3	36.4 ± 31	42.5 ± 38.1	0.06
Early MNT, < 48 h, n (%)		74.9% (393)	77.6% (128)	75.2% (158)	71.3% (107)	0.43
Kcal/kg/day*, mean ± SD		19 ± 5.6	23.1 ± 6	18.6 ± 3.7	15.27 ± 4.24	**0.001 [A]**
Protein, g/kg/day*, mean ± SD		1 ± 0.4	1.2 ± 0.4	1 ± 0.3	0.8 ± 0.2	**0.01 [A, B]**
EN		63.2% (332)	59.4% (98)	64.3% (135)	66% (99)	0.34
PN		15.4% (81)	13.3% (22)	16.2% (34)	16.7% (25)	0.85
EN-PN		7.8% (41)	8.5% (14)	7.6% (16)	7.3% (11)	0.92
PN-EN		13.5% (71)	18.8% (31)	11.9% (25)	10% (15)	0.27
**EN-related complications**						
Any complication		23.2% (122)	20.6% (34)	23.8% (50)	25.3% (38)	0.12
High GRV		12.4% (65)	11.5% (19)	13.3% (28)	12% (18)	0.54
Diarrhea		9% (47)	6.7% (11)	11.4% (24)	8% (12)	0.18
Vomiting		1.5% (8)	1.8% (3)	1.4% (3)	1.3% (2)	0.99

Aspiration	0.2% (1)	0	0.5% (1)	0	0.99
Mesenteric ischemia	0.9% (4)	0.6% (1)	0.9% (2)	0.6% (1)	0.95
**Outcomes**					
Mechanical ventilation, n (%)	92.8% (487)	89.1% (147)	93.8% (197)	95.3% (143)	0.08
Mechanical ventilation, days, mean ± SD	15.1 ± 16	13.5 ± 12.3	15.1 ± 13.7	16.7 ± 21.4	0.09
Vasoactive drug support, n (%)	77% (404)	73.9% (122)	79.5% (167)	76.7% (115)	0.44
Renal replacement therapy, n (%)	16.6% (87)	16.4% (27)	12.9% (27)	22% (33)	0.07
Respiratory tract infection, n (%)	25.3% (133)	28.5% (47)	22.4% (47)	26% (39)	0.39
Catheter-related infections, n (%)	5.9% (31)	6.7% (11)	3.8% (8)	8% (12)	0.22
ICU stay, days, mean ± SD	20.3 ± 18	18.2 ± 13.8	21.1 ± 17.1	21.6 ± 22.5	0.08
Hospital stay, days, mean ± SD	39.1 ± 32.5	40.9 ± 39	39.8 ± 29.7	36 ± 27.8	0.18
ICU mortality, n (%)	24.4% (128)	24.8% (41)	26.2% (55)	21.3% (32)	0.73
28-day mortality, n (%)	26.7% (140)	29.1% (48)	27.1% (57)	23.3% (35)	0.51

AMI: Acute myocardial infarction; COPD: Chronic obstructive pulmonary disease; PN: Parenteral Nutrition; EN: Enteral Nutrition; SD: standard deviation; APACHE II: Acute Physiology and Chronic Health Disease Classification System II; SAPS: Simplified Acute Physiology Score; SOFA: Sequential Organ Failure Assessment; SGA: Subjective Global Assessment; mNUTRIC: modified Nutrition Risk in the Critically Il; ICU: Intensive Care Unit. * During the entire administration of nutrition therapy or at least the first 14 days. Statistically significant *p*-values are written in bold. Statistical results correspond to ANOVA p values. Bonferroni post hoc testing with statistically significant differences: [A] between Normal weight and obese subgroup; [B] between overweight and obese subgroup.

Table S3. General characteristics, nutritional therapy, and outcomes of patients with obesity admitted to the ICU based on mean energy delivery categories.

		All obese *n* = 150	<11 Kcal/Kg/d *n* = 46	≥11 Kcal/Kg/d *n* = 104	p-Value
**Baseline characteristics & comorbidities**					
Age, years, mean ± SD		62.7 ± 13.5	60.07 ± 12.79	63.84 ± 13.7	*0.05*
Gender, male patients, n (%)		59.3% (89)	63.0% (29)	57.7% (60)	*0.66*
Hypertension, n (%)		56.7% (85)	54.3% (25)	57.7% (60)	*0.84*
Diabetes mellitus, n (%)		36% (54)	36.9% (17)	35.6% (37)	*0.99*
COPD, n (%)		17.3% (26)	23.9% (11)	14.4% (15)	*0.24*
AMI, n (%)		16.7% (25)	15.2% (7)	17.3% (18)	*0.94*
Chronic liver disease, n (%)		6.7% (10)	10.9% (5)	4.8% (5)	*0.18*
Chronic renal failure, n (%)		12% (18)	8.7% (4)	13.5% (14)	*0.58*
Immunosuppression, n (%)		7.3% (11)	6.5% (3)	7.7% (8)	*0.99*
Neoplasia, n (%)		18% (27)	6.5% (3)	23.1% (24)	***0.03***
Type of patient	Medical, n (%)	63.3% (95)	60.9% (28)	64.4% (67)	*0.81*
	Trauma, n (%)	10.7% (16)	17.4% (8)	7.7% (8)	*0.09*
	Surgery, n (%)	26% (39)	21.7% (10)	27.8% (29)	*0.55*
**Prognosis ICU scores & nutrition status on ICU admission**					
APACHE II, mean ± SD		21.2 ± 8.5	22.2 ± 8.8	20.8 ± 8.4	*0.18*
SAPS II, mean ± SD		52 ± 18.7	51 ± 18.2	52.4 ± 19	*0.34*
SOFA, mean ± SD		7.6 ± 3.5	8.2 ± 3.7	7.3 ± 3.4	*0.08*
Malnutrition (based on SGA), n (%)		33.3% (50)	28.9% (13)	35.6% (37)	*0.23*
mNUTRIC score, mean ± SD		4.4 ± 2	4.2 ± 2.2	4.5 ± 2	*0.30*
Patient at risk (based on mNUTRIC), n (%)		48.7% (73)	50% (22)	50.5% (51)	*0.96*
**Characteristics of Medical Nutrition Therapy**					
Time of MNT initiation, h, mean ± SD		42.5 ± 38.1	48.7 ± 39.7	39.7 ± 37.2	*0.18*
Early MNT, < 48 h, n (%)		71.3% (107)	65.2% (30)	74.1% (77)	*0.36*

EN	66% (99)	84.8% (39)	57.7% (60)	***0.002***
PN	16.7% (25)	4.3% (2)	22.1% (23)	***0.01***
EN-PN	7.3% (11)	8.7% (4)	6.7% (7)	*0.74*
PN-EN	10% (15)	2.2% (1)	13.5% (14)	***0.04***
**EN-related complications**				
Any complication	25.3% (38)	30.4% (14)	23.1% (24)	*0.08*
High GRV	12% (18)	15.2% (7)	10.6% (11)	*0.14*
Diarrhea	8% (12)	8.7% (4)	7.7% (8)	*0.95*
Vomiting	1.3% (2)	0	1.9% (2)	*0.89*
Mesenteric ischemia	0.6% (1)	2.1% (1)	0	*0.85*
**Outcomes**				
Mechanical ventilation, n (%)	95.3% (143)	100% (46)	93.3% (97)	*0.17*
Mechanical ventilation, days, mean ± SD	16.7 ± 21.4	13.41 ± 17.23	18.25 ± 23.09	*0.08*
Vasoactive drug support, n (%)	76.7% (115)	71.7% (33)	78.8% (82)	*0.46*
Renal replacement therapy, n (%)	22% (33)	15.2% (7)	25% (26)	*0.18*
Respiratory tract infection, n (%)	26% (39)	23.9% (11)	26.9% (28)	*0.85*
Catheter-related infections, n (%)	8% (12)	8.7% (4)	7.7% (8)	*0.99*
ICU stay, days, mean ± SD	21.6 ± 22.5	17.1 ± 19.3	23.7 ± 23.6	**0.04**
Hospital stay, days, mean ± SD	36 ± 27.8	28.6 ± 26.1	39.3 ± 28.1	**0.01**
ICU mortality, n (%)	21.3% (32)	32.6% (15)	16.5% (17)	**0.02**
28-day mortality, n (%)	23.3% (35)	34.8% (16)	18.7% (19)	**0.03**

AMI: Acute myocardial infarction; COPD: Chronic obstructive pulmonary disease; PN: Parenteral Nutrition; EN: Enteral Nutrition; SD: standard deviation; APACHE II: Acute Physiology and Chronic Health Disease Classification System II; SAPS: Simplified Acute Physiology Score; SOFA: Sequential Organ Failure Assessment; SGA: Subjective Global Assessment; mNUTRIC: modified Nutrition Risk in the Critically Il; ICU: Intensive Care Unit. * During the entire administration of nutrition therapy or at least the first 14 days. Statistically significant p-values are written in bold.

Table S4. General characteristics, nutritional therapy, and outcomes of patients with obesity admitted to the ICU based on mean protein delivery categories.

		All obese n = 150	<0.8 g/Kg/day n = 95	≥0.8 - <1.3 g/Kg/day n = 47	≥1.3 g/Kg/d n = 8	p-Value**
**Baseline characteristics & comorbidities**						
Age, years, mean ± SD		62.7 ± 13.5	60.1 ± 13.1	67 ± 12.5	67.5 ± 17.8	**0.01**
Gender, male patients, n (%)		59.3% (89)	57.9% (55)	65.9% (31)	37.5% (3)	0.28
Hypertension, n (%)		56.7% (85)	53.7% (51)	59.6% (28)	75% (6)	0.48
Diabetes mellitus, n (%)		36% (54)	34.7% (33)	38.3% (18)	37.5% (3)	0.91
COPD, n (%)		17.3% (26)	18.9% (18)	14.9% (7)	12.5% (1)	0.87
AMI, n (%)		16.7% (25)	15.8% (15)	19.1% (9)	12.5% (1)	0.86
Chronic liver disease, n (%)		6.7% (10)	7.4% (7)	6.4% (3)	0	0.99
Chronic renal failure, n (%)		12% (18)	12.6% (12)	10.6% (5)	12.5% (1)	0.99
Immunosuppression, n (%)		7.3% (11)	9.5% (9)	4.3% (2)	0	0.55
Neoplasia, n (%)		18% (27)	10.5% (10)	31.9% (15)	25% (2)	**0.004**
Type of patient	Medical, n (%)	63.3% (95)	64.2% (61)	61.7% (29)	62.5% (5)	0.96
	Trauma, n (%)	10.7% (16)	13.7% (13)	6.4% (3)	0	0.36
	Surgery, n (%)	26% (39)	22.1% (21)	31.9% (15)	37.5% (3)	0.36
**Prognosis ICU scores & nutrition status on ICU admission**						
APACHE II, mean ± SD		21.2 ± 8.5	21.1 ± 8.3	22.6 ± 8.5	14.8 ± 8.9	0.14

SAPS II, mean ± SD	52 ± 18.7	50 ± 17.6	57 ± 20.4	45.6 ± 17	0.18
SOFA, mean ± SD	7.6 ± 3.5	7.5 ± 3.5	8.1 ± 3.5	5.3 ± 3.4	0.12
Malnutrition (based on SGA), n (%)	33.3% (50)	29.8% (28)	36.2% (17)	62.5% (5)	0.16
mNUTRIC score, mean ± SD	4.4 ± 2	4.1 ± 2.1	4.9 ± 1.8	4.4 ± 1.7	0.12
Patient at risk (based on mNUTRIC), n (%)	48.7% (73)	45.6% (42)	63.1% (29)	25% (2)	0.07
**Characteristics of Medical Nutrition Therapy**					
Time of MNT initiation, h, mean ± SD	42.5 ± 38.1	42.3 ± 39.2	43.1 ± 33.6	40.6 ± 52.9	0.98
Early MNT, < 48 h, n (%)	71.3% (107)	72.6% (69)	68.1% (32)	75% (6)	0.90
EN	66% (99)	75.8% (72)	53.2% (25)	25% (2)	**0.001**
PN	16.7% (25)	12.6% (12)	19.1% (9)	50% (4)	**0.03**
EN-PN	7.3% (11)	6.3% (6)	10.6% (5)	0	0.64
PN-EN	10% (15)	5.2% (5)	17% (8)	25% (2)	**0.02**
**EN-related complications**					
Any complication	25.3% (38)	26.3% (25)	27.6% (13)	50% (4)	0.36
High GRV	12% (18)	7.4% (7)	12.7% (7)	50% (4)	0.08
Diarrhea	8% (12)	9.5% (9)	6.4% (3)	0	0.87
Vomiting	1.3% (2)	2.1% (2)	0	0	0.99
Mesenteric ischemia	0.6% (1)	0	(1)	0	0.99
**Outcomes**					
Mechanical ventilation, n (%)	95.3% (143)	100% (95)	91.5% (43)	62.5% (5)	0.45
Mechanical ventilation, days, mean ± SD	16.7 ± 21.4	14.5 ± 16.7	22.3 ± 29.4	7.8 ± 6.6	0.09
Vasoactive drug support, n (%)	76.7% (115)	73.7% (70)	80.8% (38)	87.5% (7)	0.60
Renal replacement therapy, n (%)	22% (33)	22.1% (21)	23.4% (11)	12.5% (1)	0.94
Respiratory tract infection, n (%)	26% (39)	26.3% (25)	23.4% (11)	37.5% (3)	0.66
Catheter-related infections, n (%)	8% (12)	8.4% (8)	8.5% (4)	0	0.99
ICU stay, days, mean ± SD	21.6 ± 22.5	19.5 ± 17.8	27.6 ± 30.4	12.4 ± 5.1	0.06
Hospital stay, days, mean ± SD	36 ± 27.8	33 ± 27.6	40.8 ± 26.1	43.4 ± 38.7	0.23
ICU mortality, n (%)	21.3% (32)	25.5% (24)	14.9% (7)	12.5% (1)	**0.02**
28-day mortality, n (%)	23.3% (35)	25.2% (24)	21.3% (10)	12.5% (1)	0.07

AMI: Acute myocardial infarction; COPD: Chronic obstructive pulmonary disease; PN: Parenteral Nutrition; EN: Enteral Nutrition; SD: standard deviation; APACHE II: Acute Physiology and Chronic Health Disease Classification System II; SAPS: Simplified Acute Physiology Score; SOFA: Sequential Organ Failure Assessment; SGA: Subjective Global Assessment; mNUTRIC: modified Nutrition Risk in the Critically Il; ICU: Intensive Care Unit. ** Statistically significant differences between between inadequate (i.e., <0.8 g/Kg/day) and insufficient (0.8 - <1.3 g/Kg/day) protein delivery subgroups.
